# Intra-arrest-cooling PRO

**DOI:** 10.1186/1471-227X-15-S1-A11

**Published:** 2015-06-24

**Authors:** Fabio Silvio Taccone

**Affiliations:** 1 Department of Intensive Care, Hopital Erasme, Université Libre de Bruxelles, Brussels, Belgium

## 

Although targeted temperature management (TTM) has been widely implemented among comatose survivors after cardiac arrest (CA), there are still several unanswered issues, including the optimal time to initiate cooling. Experimental studies have showed that early cooling after return of spontaneous circulation (ROSC) provides better neurological protection than normothermia, while clinical studies failed to show any significant benefits of this strategy [1]. Importantly, several experimental data suggested that hypothermia initiated during cardiopulmonary resuscitation (CPR) – that is, so-called intra-arrest hypothermia (IAHT) – increased the effectiveness of resuscitation attempts and defibrillation when compared with normothermia [2,3]. In a recent systematic review, Scolletta and colleagues identified 23 animal studies and five human studies which evaluated the effects of IAHT in this setting [4]. In particular, animal studies showed that IAHT improved survival and neurological outcomes when compared with normothermia and/or hypothermia after ROSC. IAHT was also associated with improved ROSC rates and with improved cardiac function, including better left ventricular function and reduced myocardial infarct size, when compared with normothermia.

Unfortunately, clinical data on the efficacy of IAHT remain limited. In a retrospective study, Garrett and colleagues compared the outcome for 208 out-of-hospital CA patients treated with intra-arrest cold intravenous fluids with historical controls ( *n*= 334) [5]. The use of IAHT was associated with an increased ROSC rate, while it could not improve overall survival to hospital admission or to discharge. Nevertheless, less than 10% of patients admitted to the hospital were eventually treated with in-hospital TTM, which negatively influenced the effects of early cooling on patients’ outcome. In a recent randomized clinical trial, Debaty and colleagues evaluated the effects of intra-arrest cold fluids when compared with TTM started after hospital admission on out-of-hospital CA patients, irrespective of their initial rhythm [6]. Of the 245 patients included (*n* = 123 in the IAHT group; *n* = 122 in the control group), IAHT significantly reduced the time to reach body temperature below 34°C by 75 minutes; however, the proportion of patients admitted alive to hospital was not different between groups (33% vs. 30%; *P* = 0.51). Levels of neuron-specific enolase, a biomarker of brain injury, which was considered the primary outcome of the study, were not different between groups and no difference in survival and 1-month neurological recovery was found. Importantly, intra-arrest cold fluids are potentially associated with important adverse events, such as a reduced coronary perfusion pressure, a longer duration of CPR and the development of lung edema, which may have blunted their beneficial effects [7].

In another randomized clinical trial using intra-arrest transnasal evaporative cooling [8], out-of-hospital CA patients were randomized, irrespective of their rhythm, to receive IAHT (*n* = 96) or standard of care ( *n*= 104, including in-hospital TTM) during CPR [8]. Overall survival rates were similar in the two groups (15% vs. 13%). Among patients admitted to the hospital, overall survival was increased, although not significantly, from 31 to 44% using IAHT (*P* = 0.16). Also, IAHT increased, although not significantly (*P* = 0.14), the intact neurological outcome rate from 21% to 34% when compared with controls; these beneficial effects were more pronounced in patients with short time to CPR (that is, <10 minutes; 43% vs. 17%, *P*= 0.03). An ongoing randomized clinical trial will include nearly 800 patients to confirm these promising preliminary results using intra-arrest transnasal evaporative cooling in out-of-hospital CA.

## Financial disclosure

FST has a research grant from Benechill in 2010 for animal research on IAHT.

## Declaration

This abstract and supplement was proposed, developed and commissioned by BMC Emergency Medicine and was funded by an educational grant from C. R. BARD, NJ, USA. The published abstract was independently prepared by the author. C. R. BARD had no input into the content.
